# Serum Selenium Level in Early Healthy Pregnancy as a Risk Marker of Pregnancy Induced Hypertension

**DOI:** 10.3390/nu11051028

**Published:** 2019-05-08

**Authors:** Małgorzata Lewandowska, Stefan Sajdak, Jan Lubiński

**Affiliations:** 1 Division of Gynecological Surgery, Poznań University of Medical Sciences, 60-535 Poznań, Poland; ssajdak@ump.edu.pl; 2 Department of Genetics and Pathology, International Hereditary Cancer Center, Pomeranian Medical University, 71-252 Szczecin, Poland; lubinski@pum.edu.pl

**Keywords:** hypertension, pregnancy, selenium, BMI, smoking, risk, micronutrient, prospective study

## Abstract

Selenium (Se) is an antioxidant nutrient whose deficiency can influence adverse outcomes of pregnancy. The aim of this study is to determine whether serum Se level in early healthy pregnancy may be a risk marker for pregnancy induced hypertension. We obtained data from our prospective study in which we recruited healthy women in weeks 10–14 of a single pregnancy. In this analysis, we examined 121 women who subsequently developed pregnancy-induced hypertension and matched 363 women who remained normotensive. We measured Se levels (using the ICP-MS technique) in the serum in weeks 10–14 of the pregnancy. The odds ratios of pregnancy-induced hypertension (95% confidence intervals) were calculated using multivariate logistic regression. We found that the mean Se level was lower in the case group compared to the control (57.51 vs. 62.89 μg/L; *p* = 2.6 × 10^−10^). Excessive body mass index (BMI) and smoking influenced the estimated odds ratios. In the subgroup of women who had never smoked with normal pre-pregnancy BMI, the adjusted odds ratio (AOR) of pregnancy-induced hypertension was 15.34 (95% CI: 2.73–86.31, *p* = 0.002) for Se levels in the lowest quartile (≤57.68 µg/L), as compared to the highest quartile (>66.60 µg/L), after adjusting for all the accepted confounders. In the whole cohort, the prognostic value of Se by logistic regression showed that the area under curve (AUC) = 0.814. In our study, one can consider the role of Se as a risk marker of pregnancy-induced hypertension.

## 1. Introduction

Micronutrient deficiencies can influence pregnancy outcomes [1]. Selenium (Se) is a micronutrient of great importance for human health. Numerous studies have shown that the deficiency of this element may be associated with cardiovascular diseases, some cancers, the degeneration of articular cartilage, diseases resulting from reduced immunity, thyroid dysfunctions, and the improper functioning of the nervous system, among others. Se deficiencies have also been associated with negative effects on embryo development [2,3,4]. Nowadays, researchers are working on new strategies for the normalization of this microelement level [2,3,5] and the possibilities of using Se in the creation of new drugs [6,7].

Selenium is a trace element which is important for numerous biological processes. It is involved, inter alia, in the antioxidant defense system, in the functions of the immune system, in the development of the inflammatory reaction, in apoptosis, and in detoxification processes [4,8,9]. Selenium performs its functions, being included (as selenocysteine) in the active sites of proteins called selenoproteins [10]. The presence of such selenoproteins as antioxidative glutathione peroxidase (GPx), and thioredoxin reductase (Th-red)—among others—are expressed in the uterus [11].

A low level of Se is typical for the inhabitants of Central Europe. Epidemiological studies have found an association between low levels of this microelement and occurrence of preeclampsia in many countries, including Poland [12].

Pregnancy-induced hypertension is a disease characterized by the development of arterial hypertension de novo after the 20th week of pregnancy, and it is one of the main causes of maternal and fetal mortality. It affects an average of 5–10% of pregnant women, and it includes preeclampsia (PE) and isolated gestational hypertension (GH) [13]. Risk factors include chronic hypertension, pre-pregnancy diabetes, mother′s age, primipara, obesity, and smoking [14]. The disease etiology is not fully understood, and the key significance of oxidative stress is emphasized in its pathogenesis; it has been found that early trophoblast invasion disorders result in hypoxia of the placenta and intensification of oxidative stress, activation of the inflammatory response, and a cascade of disorders, often occurring in a vicious circle mechanism. The result is damage to the structure and function of the endothelium. This results in an increase in blood pressure de novo after the 20th week of pregnancy [13,15,16].

In numerous studies in women with preeclampsia, a statistically significant relationship was found with lower levels of Se and lower levels of glutathione peroxidase (GPx), thioredoxin reductase (Th-red), and anti-inflammatory selenoprotein SEPP1 in the placenta or serum/plasma, compared to those of healthy pregnant women [17,18,19,20]. However, not all results are unambiguous [21]. Selenium levels in women with preeclampsia may be the result of already-developed disorders.

The problem remains open as to whether this microelement deficiency in early pregnancy, in healthy women in the first half of pregnancy, may be related to the subsequent development of pregnancy-induced hypertension [22]. Prospective studies are needed to solve this problem; such studies are few in number, their methodologies are different, and their results are divergent [22,23,24,25].

The reason for the discrepancy may be the use of different clinical and biochemical methodologies. The choice of biological material to measure Se levels is also important. Examining critically ill patients with a systemic inflammatory response, Stefanowicz et al. found correlations between inflammation and Se levels in the plasma but not in erythrocytes [26]. Inflammation can also affect the differences between serum and plasma in concentrations of many compounds [27]. Assessment of the Se status in the serum is likely to provide the best possibility of detecting changes.

The aim of our study was to determine whether serum selenium level in early healthy pregnancy may be a risk marker of pregnancy-induced hypertension. As far as we know, this is the first single-center study for serum levels in this element, conducted for such a great number of cases. In our study, one can consider the role of Se as a risk marker of pregnancy-induced hypertension.

## 2. Materials and Methods

The study was conducted in accordance with the Helsinki Declaration; all the participants signed the Informed Consent Form and the Test Information Form before submitting a blood sample. The study was approved by the Bioethics Committee of the Medical University of Poznan, Poland, under number 769/15.

This analysis was conducted in accordance with the guidelines for study designs (from EQUATOR network).

### 2.1. Population and Design

We obtained data from our prospective cohort study. We conducted this study at the University Hospital in Poznan, Poland (third-degree reference center, with 6–8 thousand births a year). We conducted the recruitment among pregnant women taking typical laboratory tests. The women were observed until the postpartum period. The recruitment was conducted in 2015–2016. The observation and analyses were conducted in 2016–2017 and 2017–2019, respectively.

The participants were selected based on the following criteria: Women of white Polish descent (Central Europe) from the Wielkopolska region, healthy, aged 18–45 years at conception, in week 10 (+0)–14 (+6) of pregnancy (confirmed single pregnancy running its course correctly without aneuploidy and with subsequent delivery of a phenotypically normal child ≥25 weeks of pregnancy), no chronic diseases apart from being overweight or obese (in particular, no chronic hypertension, pre-pregnancy diabetes mellitus, kidney and liver diseases, immunological and inflammatory diseases, and thromboembolism), no active infections, the use of normal diet. The use of vitamin preparations for pregnant women was not a condition of inclusion in the study. The women were observed until to the 12th week after parturition.

At baseline, 1300 women were recruited through advertisements. The women who did not meet all the inclusion criteria after the observation period ended (n = 48), as well as those whose data were incomplete (n = 340), were excluded. In 16 cases, the serum was unavailable. After the exclusion, we qualified 896 women for this study (121 women who developed pregnancy-induced hypertension and 775 women who remained normotensive). After matching the participants, we examined 484 women in two groups. The study group (n = 121) included the women who subsequently developed pregnancy-induced hypertension (106 cases of gestational hypertension and 15 cases of preeclampsia), and the control group (n = 363) comprised the women who remained normotensive.

The sample size was calculated using the following formula for a single proportion:

*n* = *Z*^2^/*d*^2^ × *p*(1 − *p*) (1)

(“*Z*”–critical value of the normal distribution at α/2, α = 5%, confidence intervals = 95%, *Z* = 1.962; “*p*”–sample proportion; “*d*”–margin of error). For the proportion of *p* = 10% (based on the literature) [13] and the margin error *d* = 2%, the estimated sample size (for whole studied population) was 864.

For the proportion of *p* = 14% (based on our population; 121 cases/896 = 13.51%) and the margin error d = 3%, the estimated sample size was 514.

**Scheme 1:**
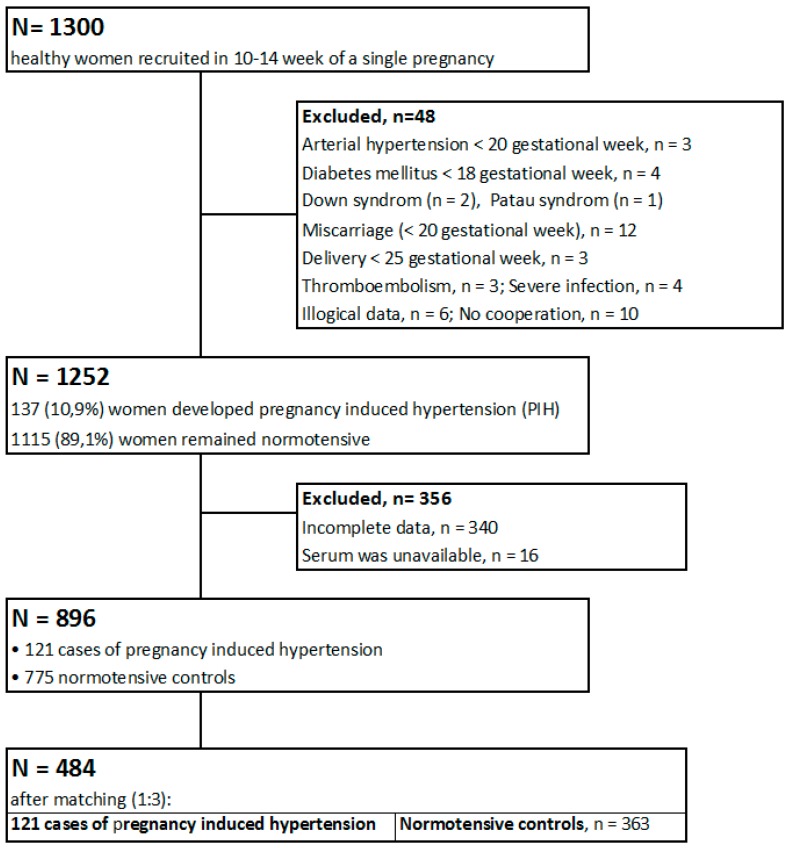
The recruitment scheme.

The recruitment results are presented in Scheme 1.

### 2.2. Method and Data Collection

Socio-demographic and anthropometric characteristics, clinical data (obstetrical and gynecological histories, medications, and multivitamins), and other information (family history, smoking and alcohol consumption during pregnancy) were collected using a personal questionnaire during the recruitment. The participants themselves filled out survey forms (but in the presence of midwives) so the influence of the interviewers on the answers could be excluded. The women were observed up to 12 weeks after parturition; contact with the participants (telephone, e-mail) was maintained. Pregnancy and neonatal outcomes were taken from the medical records, and some information was passed on by the participants themselves; the information was verified several times during the observation.

The data included the following maternal education level categories: Elementary, vocational, secondary, and higher. The financial status was assessed according to a 5 Lickert′s scale, on the basis of an answer to the question: “Is your financial situation (in your household) good enough to meet your needs?” The responses were classified in the following way: (1) definitely NO; (2) rather NO; (3) hard to say; (4) rather YES; (5) definitely YES. In this survey we distinguished lower financial levels (1 and 2), medium (3), and higher (4 and 5). Data concerning the place of residence included the following categories: Countryside, small town (<50 thousand inhabitants), and big city.

All women declared no alcohol in pregnancy. Self-reported pre-pregnancy weight was collected from participants. The normal pre-pregnancy BMI (body mass index) was defined as: 18.5–24.99 kg/m^2^. Gestational weight gain (GWG) was calculated as the difference between the weight measured before delivery (as recorded in the medical records) and the pre-pregnancy weight.

All participants declared normal blood pressure (< 140/90 mmHg) before pregnancy. The blood pressure was measured in a sitting position with an oscillometric device on the arm. We recorded the first blood pressure measurement in Medical Records before recruitment. After parturition, we recorded the blood pressure taken in the maternity ward after leaving the postpartum ward.

Pregnancy-induced hypertension was defined in accordance with the national guidelines (2015) convergent with the new definition of preeclampsia [14] as “arterial pressure equal to and higher than 140/90 mmHg (on two occasions, at least 4 h apart) developed de novo after the 20th week of pregnancy, receding up to 12 weeks after delivery.” The disease includes preeclampsia (PE) and isolated gestational hypertension (GH). Gestational hypertension was diagnosed if no other disturbance was found. “Preeclampsia was diagnosed when any of the following appeared de novo: proteinuria (≥300 mg/day or ≥0.3 g/L, protein/creatinine ratio ≥0.3; 1+ in the strip test), thrombocytopenia <100 G/L, worsening of renal function; damage to the liver function; pulmonary edema; or symptoms from the central nervous system; blurred vision”. In our study, only proteinuria (≥300 mg/L) occurred in the cases of preeclampsia. IUGR (Intrauterine Growth Restriction) was not a criteria of diagnosis.

Before the study, clinical risk factors of pregnancy-induced hypertension [14] and influencing factors related to the level of Se [1] were identified based on literature data.

### 2.3. Serum Selenium Determination

Blood samples were taken (in the 10–14th gestational week) by venipuncture using the Sarstedt Monovette system (Sarstedt, Germany) with Serum Z/7.5 mL tubes. After donation, blood was left to clot for at least 30 min. Samples were centrifuged for serum separation (1300 G, 12 min) for 120 min after donation. After that, the serum was transferred into cryo vials and placed into freezer at −80 °C until analysis. At the day of analysis, samples were thawed, vortexed, and centrifuged at 5000 G for 5 min before Se determination.

We examined selenium levels in the serum by the inductively coupled plasma mass spectrometry (ICP-MS) method. Sample total Se determination was performed on an ICP mass spectrometer NexION 350D (PerkinElmer, Shelton, CT, USA). The spectrometer was equipped with a dynamic reaction cell (DRC) operating with high purity methane and was calibrated using external calibration technique.

Calibration standards were prepared from a 10 µg/mL Multi-Element Calibration Standard 3 (PerkinElmer, Shelton, CT, USA) by diluting with a blank reagent to the final concentration of 0.1; 0.5; 1.0; 2.0; 5.0; 10 µg/L. Correlation coefficients for calibration curves were always greater than 0.999.

Analysis protocol assumed a 100-fold dilution of serum in blank reagent. A blank reagent consists of 10 mL of 65% Suprapur Grade nitric acid (Merck, Darmstadt, Germany) and 0.20 mL of Triton X-100 (PerkinElmer, Shelton, CT, USA) filled to the mark of 1 L flask with class I deionized water (Merck Millipore). Germanium isotope (Ge74) was set as internal standard. The accuracy and precision of measurements were tested using Certified Reference Material (CRM), Clincheck Plasmonorm Serum Trace Elements Level 1 (RECIPE, Munich, Germany).

Additionally, internal quality control samples were measured during analysis. The general precision was lower than 5% RSD (Relative Standard Deviation). The final concentration included a dilution factor and coefficient, which was the mean value of two flanking certified reference material concentrations divided by the mean concentration determined by the manufacturer of CRM.

### 2.4. Statistical Analyses

The data were imported into the Statistica 13 package in order to perform calculations. The data were compared between the cases and the control group. The normality of the data distribution in groups was checked by the Shapiro-Wilk test. The Mann-Whitney U test was used for comparisons of continuous variables (variables were not normally distributed), and the Pearson chi-square test was used for categorical variables comparisons (*p*-value <0.05 was assumed to be significant). Serum Se concentrations were compared between groups using the Mann-Whitney U test (selenium levels were not normally distributed; medians were compared).

We did individual matching. We chose the control normotensive group by matching cases of pregnancy-induced hypertension (in a 1:3 ratio) in relation to the following criteria: Mother′s age, pre-pregnancy BMI, and those who have never smoked. Due to the inability to select women at exactly the same age, we had to expand the selection by ±2 years. We found the difference in the maternal age between the groups (*p* = 0.907). For this reason, we did not use the conditional logistic regression; we used logistic regression.

As confounders, we used gestational age during recruitment, which may affect selenium levels (statistically significantly different between groups) and risk factors for pregnancy induced hypertension, which statistically significantly differed between groups: The pre-pregnancy BMI, gestational weight gain (GWG) for one week of pregnancy (calculated for whole gestation), family history of chronic hypertension, and maternal education level <12 years. The analyses were carried out for the whole cohort (N = 484) and the subgroup of women who had never smoked with normal pre-pregnancy BMI separately (N = 228). In the subgroup, the pre-pregnancy BMI was excluded.

The whole cohort and the subgroup were divided into quartiles. The odds ratios (and 95% confidence intervals CI) of pregnancy-induced hypertension were calculated by univariate (OR-crude) and multivariate (AOR) logistic regression for serum Se concentrations in each quartile with respect to the quartile with the highest the number of normotensive females (OR = 1.00). *p*-value was calculated using the Wald test, and value <0.05 was assumed to be significant. We adjusted the risk for all the accepted confounders. Graphs showing the risk profiles were presented.

Predictive indicators of pregnancy-induced hypertension for Se levels for false positive rates (FPR) of 5% and 10% were calculated using logistic regression and a neural network. In both methods, a test set that comprised 20% of the total data set was randomly selected, while the remaining 80% served as a training data set.

## 3. Results

The clinical characteristics of the cases and normotensive groups are presented in Table 1. The differences were statistically insignificant in terms of maternal age, parity, number of those who had never smoked, pack-years in smokers, methods of assisting reproduction, gestational diabetes mellitus, and use of multivitamins during pregnancy.

The mean age of women at conception was 35.05 years in the cases group (range 19–45) and 35.05 years in the normotensive group (range 22–45) (*p* = 0.907). We found 46.3% of primiparous in the cases group and 38.8% in the control group (*p* = 0.149). In the cases group, compared to the normotensive women matched, the mean pre-pregnancy BMI was higher (26.76 kg/m^2^ vs. 25.03 kg/m^2^; *p* = 0.003), and the mean gestational age during recruitment was lower (11.55 vs. 12.25 week; *p* = 1.97·× 10^−16^).

In the socioeconomic characteristics, we found differences in education level categories (*p* = 0.042), financial status level categories (*p* = 0.002), and place of residence (*p* = 0.585). In the case group, we found more frequent occurrence of elementary and vocational education levels, less frequent occurrence of high financial status, and more frequent residence in the big city, compared to the normotensive control group.

The recruitment results are presented in Scheme 1 (in chapter of Materials and Methods).

Serum Se levels in the groups and subgroups are presented in Table 2 and Table S1. In the whole cohort, the mean microelement level in the 10–14th pregnancy week was lower in the women subsequently developing pregnancy-induced hypertension than in the matched normotensive women (57.51 vs. 62.89 μg/L; medians 57.40 vs. 62.02, *p* = 2.59 ×·10^−10^).

The average Se level was lower in the women with the pre-pregnancy BMI ≥25 kg/m^2^ than in the women with the BMI within the normal range (60.27 vs. 62.23 μg/L; medians 58.91 vs. 61.57, *p* = 0.0007) and lower in the smokers at the time of recruitment compared to the women who had never smoked (57.74 vs. 61.91 μg/L; medians 57.62 vs. 61.22, *p* = 0.018).

**Figure 1:**
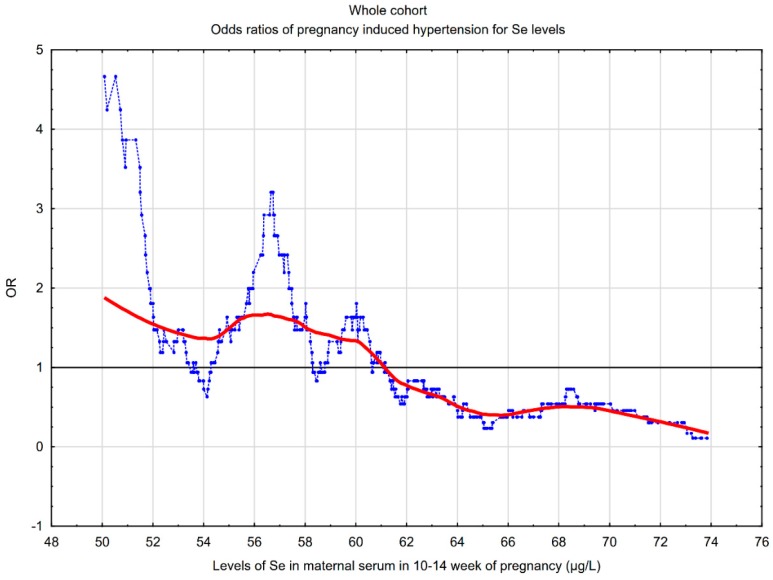
The risk of pregnancy-induced hypertension for selenium levels in the 10–14th pregnancy week in the whole cohort. The graph illustrates the changes in the odds ratios (OR) of pregnancy-induced hypertension (PIH), calculated on a sliding window with respect to the changes in the Se levels in serum in the 10–14th pregnancy week. The window width adopted was 50 observations. The (light blue) points correspond to the odds ratios of pregnancy-induced hypertension in a window containing a fixed number of neighboring cases whose center is for a Se level value. The (red) curve represents the risk profile smoothed with the Lowess method. The horizontal (black) line marks is the reference line for OR = 1.0; the points above this line indicate an increased risk, and the points below this line correspond to a reduction in risk.

The graphical picture of risk profile of pregnancy-induced hypertension for Se levels in the whole cohort is presented in Figure 1.

The pictures illustrate that lower levels of this element in serum in the 10–14th week of pregnancy are associated with a higher risk of the disease.

The risk profile in the subgroup of women who had never smoked with normal pre-pregnancy BMI is presented in Figure S1.

The risk of pregnancy-induced hypertension after having divided the whole cohort into quartiles according to the distribution of the Se levels is shown in Table 3. We found that the number of cases of pregnancy-induced hypertension increased along with decreasing levels of Se in serum in the 10–14th gestational week.

In the lowest Q_1_ quartile, the highest number of cases was found (47 cases among 121 women). In the highest Q_4_ quartile, the lowest number of cases was found (13 cases among 121 women). The adjusted odds ratio (AOR) of pregnancy induced hypertension was 3.96 (95% CI: 1.87–8.42, *p* = 0.0003) for serum Se levels in the lowest quartile (≤55.83 μg/L), compared to the highest quartile (>65.82 µg/L) after being adjusted for all the accepted confounders in multivariate logistic regression.

In the subgroup of women who had never smoked with normal pre-pregnancy BMI women, the adjusted odds ratio (AOR) of pregnancy-induced hypertension was 15.34 (95% CI: 2.73–86.31, *p* = 0.002) for serum Se levels in the lowest quartile (≤57.68 µg/L), compared to the highest quartile (>66.60 µg/L) after being adjusted for all the accepted confounders in multivariate logistic regression.

The prediction indicators of pregnancy-induced hypertension for serum Se levels in the neural network and logistic regression are presented in Table 4 and Table S2. The analysis was carried out for the whole cohort (N = 484). Indicators were determined for false positive rates (FPR) of 5% and 10%. In the test sets, the prognostic value of Se in pregnancy-induced hypertension by logistic regression showed the area under curve (AUC) = 0.814 (see in Table 4). The prediction indicators in the training sets are presented in Table S2.

## 4. Discussion

The aim of this study was to determine whether the serum selenium level in early healthy pregnancy may be risk marker of pregnancy-induced hypertension. We found that the mean serum Se level in the 10–14th gestational week was statistically significantly lower in the women subsequently developing pregnancy-induced hypertension (n = 121), compared to the matched women who remained normotensive (n = 363) (*p* = 2.6 × 10^−10^). We found that the risk of pregnancy-induced hypertension increased along with decreasing levels of Se in the serum. We found that excessively high BMI and smoking have an influence on the estimated odds ratio. In the subgroup of women who had never smoked with normal pre-pregnancy BMI, the adjusted odds ratio (AOR) of this dangerous disease was 15.34 (95% CI: 2.73–86.31, *p* = 0.002) for serum Se levels in the lowest quartile, compared to the highest quartile (being adjusted for all the accepted confounders). The prognostic value of serum Se levels in the 10–14th gestational week was high (AUC = 0.814).

The strength of this study was the model of a prospective cohort study, which is the only one that allows to assess whether Se can be a marker of the risk of pregnancy-induced hypertension. An advantage was the large number of cases in the single-center study. An advantage of the study was that it had a good match between groups and risk adjustment for identified risk factors; however an impact of different confounders is possible. We used a new definition of preeclampsia. Another advantage was an additional analysis after dividing participants into subgroups with regard to smoking and a pre-pregnancy BMI, but the number of participants was lower in the subgroup of women who had never smoked with a normal pre-pregnancy BMI. We adjusted the risk in the subgroup by the same confounders as in the whole cohort study. The prediction indicators complemented the risk assessment. To our knowledge, our prospective study is the first in which we determined the prediction indicators of pregnancy-induced hypertension for this element for false positive rates (FPR) of 5% and 10%.

One limitation in our study was a small number of cases of preeclampsia. Seasonal variation of selenium levels was not accounted in our analyses. Examining the level of the microelement at several time points during pregnancy would be an interesting complement to the study. Participants reported fasting before blood samples were taken, but this may be of limited value in pregnant women because of the common impairment of peristalsis in pregnancy. The participants of the study reported some data on their own, but the most important data came from the medical records, and all the information was verified several times during the observation.

In the literature, we found only few prospective studies dealing with the assessment of this trace status in the risk of pregnancy-induced hypertension, but the results are inconsistent.

Results convergent with ours were obtained by Rayman et al.; they obtained a lower risk of pregnancy-induced hypertension for higher Se levels in the nail from the 16th gestational week [22]. Ghaemi et al. found a lower plasma level of Se in the 20–24th weeks of pregnancy in 38 primigravidae subsequently developing preeclampsia compared to 38 matched healthy primigravidae (the crude odds ratio of preeclampsia was 9.14, 95% CI: 2.25–37.01, for plasma levels <62.2 µg/L compared to >74.4 µg/L) [23]. The epidemiological report of Vanderlelie and Perkins from 45 countries from different continents showed that in countries where mean plasma or serum Se levels were higher than 95 μg/L, preeclampsia was statistically significant lower (*p* = 0.0007); Poland was among the countries with a low selenium level in the plasma (68.3 μg/L) and a high preeclampsia rate (4.4%) [12]. The predictive indices obtained in our study (area under curve AUC = 0.814) are high in comparison with the results in the literature for other clinical and biochemical predictive models [14].

Results differing from ours were obtained by Mistry et al. in a study that was part of the SCOPE (Screening for Pregnancy Endpoints) study of women with single pregnancies without a high risk of developing preeclampsia (a multi-center international prospective cohort study) [24]. The authors found statistically insignificantly lower levels of Se in the plasma at 15 ± 1 week of pregnancy in 244 women who subsequently developed preeclampsia, compared to 472 women who remained normotensive. The examined groups were matched in terms of age and BMI, but the cohort studied came from six centers from four countries. Basu et al. [25] also found statistically insignificantly lower serum levels of Se at 12.2 ± 1.9 weeks in 23 women who subsequently developed preeclampsia, compared to the results of 24 normotensive women, but the study included a cohort of 151 women with a single pregnancy with pre-pregnancy diabetes mellitus.

In contrast, numerous retrospective studies have found a statistically significant connection between low Se levels and pregnancy-induced hypertension [17,18,20,28]. However, another retrospective study does not confirm these results [21]. For example, Katz et al. [28] found a statistically significantly lower serum Se levels in 43 cases of severe preeclampsia, compared to 80 healthy pregnant women matched according to gestational age. Da Silva et al. [21] found no differing of serum Se levels in 38 women with preeclampsia, compared to 32 normotensive women.

A meta-analysis of Se supplementation studies performed by Xu et al. [29], including 13 observational studies and 3 randomized controlled trials; they showed that this supplementation reduced risk of preeclampsia. However, a systematic review of Salles et al. [30] showed no statistically significant effect of antioxidant supplementation, including Se, on the development of preeclampsia. Selenium displays a “U” curve, which means that both too low and too high Se levels are associated with an increase in morbidity [31]. Therefore, supplementation in people with normal levels of this element levels may not bring the expected effects and may even be harmful.

The discrepancies among different research results could be due to different constructs of the studies and differences in population risk, size of groups, degree of match, and confounding variables, as well as biochemical materials (determination of the microelement in the serum, plasma, nails, erythrocytes, or leukocytes).

In our study, we examined microelement levels in the serum by the inductively coupled plasma mass spectrometry (ICP-MS) method. In our methodology, we excluded a priori chronic diseases existing before pregnancy (except for being overweight and for obesity) as well as racial differences, multiple pregnancies, fetal defects, and delivery before the 25th pregnancy week. We matched the studied groups well to several clinical factors, and our study covered one geographical region of Poland, which additionally matched the researched women with respect to diet composition in the region and the same level of prenatal care. Geographical differences can cause discrepancies. The average Se level in North America is 122.4–151.8 μg/L, while the level in the serum / plasma of Poles is about 70 μg/L; in some regions, it is even 50–55 μg/L [2,32,33,34]. In our control (normotensive group), the mean Se level in the serum in the 10–14th pregnancy week was 62.89 μg/L (range 41.14–125.54 µg/L). In our study, we recruited women in a specific range of gestational age (the 10–14th pregnancy week), but the differences between groups were statistically significant, so we included this feature as confounders. Most studies show that this microelement levels in pregnancy become lower [35].

In our study, mean serum Se levels in early healthy pregnancy were lower in the women with pre-pregnancy overweight/obesity than in the women with the pre-pregnancy BMI within the normal range (*p* = 0.0007) and in smokers than in those who have never smoked (*p* = 0.018) (Table 2). For the micronutrient levels in the lowest Q1 quartile compared to the highest quartile, the adjusted odds ratio (AOR) of the disease was 3.96 (*p* = 0.0003) in the whole cohort and 15.34 (*p* = 0.002) in the subgroup of women who had never smoked with normal pre-pregnancy BMI (Table 3). Data from the literature confirm the existence of this element deficiency in obese people compared to people with a normal BMI and in smokers compared to non-smokers [36,37]. Our results suggest that changes in serum Se levels in overweight/obese women and in smokers strongly influence the estimated odds ratios and the structure of the studied populations; BMI and smoking may cause discrepancies between studies.

In our study, we also compared socio-economic factors between groups. They can affect the risk of disease and the level of the micronutrient. In our study, the group developing hypertension was characterized by a greater frequency of lower education levels. Higher financial status was less frequent in the case group. It may be connected to worse access to proper health care and medicines or multivitamins, the lack of ability to recognize symptoms of the threat, and a lack of knowledge about the harmfulness of a bad lifestyle (e.g., obesity, smoking), among others.

Methods of compensating for selenium deficiencies are the subject of the latest research [2,3]. The main source of this element is an appropriate and balanced diet. However, depending on the region of the world, there may be a need to top it up due to the intake of products that are low in Se. The research includes the use of direct supplementation (in the form of supplements containing micronutrients and vitamins) or indirect supplementation (by adding this microelement to fertilizers or animal fodder). The effects depend, among other things, on the chemical form of the micronutrient. The preferred strategy for compensating for shortages of this element are Se organic yeast preparations (containing selenomethionine) with a higher bioavailability. Inorganic Se salt (selenites and selenates) may be a more favorable solution when a rapid effect is recommended, e.g., in the treatment of tumors. Strategies for the use of functional food include inter alia, foods containing lactic acid bacteria (that accumulate Se). It is emphasized, however, that the strategies used must be proven and safe, and they require further research [2,3]. Still, other studies indicate the cytotoxic effect of excess Se, which may be conductive to the development of certain diseases but may also be used in developing new drugs that target fungi, bacteria, or cancer cells [6]. An interesting issue is the study of new anticancer drugs that use reactive oxygen species (ROS) levels by redox modulation and whose aim are selenoproteins TrxR (thioredoxin reductases) [7].

The main mechanism connecting the low serum Se level in the 10–14th pregnancy week with the risk of pregnancy-induced hypertension may be a deficiency of this micronutrient antioxidant effects in the time of trophoblast development. However, processes related to the development of the placenta are multifactorial, complicated, and not fully explained. The results of different studies indicate that under normal conditions, low oxygen tension in the placenta in the 8–10th week of pregnancy is accompanied by a low level of reactive forms of oxygen and nitrogen. Next, with the normal progression of trophoblast invasion, blood flow in the placenta increases (in the 11–12th week of pregnancy, the oxygen tension was around 50 mmHg) and reactive forms of oxygen increase [38]. However, the environment of the uterus is equipped with enzymes with antioxidant activity (selenium-dependent also) [11]. In experimental studies, the Se supplement was shown to increase the level of selenium-dependent glutathione peroxidase (GPx) in trophoblastic cells exposed to experimental prooxidant activity or to protect mitochondria of trophoblasts [39,40]. The exposure of cells to oxidative stress leads to a decrease in the expression and antioxidant activity of selenoproteins, which require adequate Se supply for their activity.

Other Se effects may also be part of the pathomechanisms of pregnancy-induced hypertension, including the element′s participation in the immune and inflammatory response, in apoptosis, and in detoxification processes [4].

It has been found that abnormal invasion of trophoblasts in the walls of the spiral arteries (between the 6–18th weeks of pregnancy) results in their insufficient re-modelling, which leads to utero-placental high-resistance circulation. This results in hypoxia and intensification of the production of reactive oxygen species, affecting apoptosis, the immune system, and the intensification of the inflammatory response. The cascade of processes in the placenta and, subsequently, in the maternal circulation system, is activated and leads to the damage of the vascular endothelium; this results in an increase in blood pressure [13]. Pathophysiological mechanisms of this disease are complex, multifactorial, and not fully explained (metabolic, genetic, and immunological). However, in the circulatory system of women with advanced preeclampsia, the presence of markers released by activated or damaged endothelium (markers of oxidative stress, von Willebrand factor, thrombomodulin, endothelin-1, fibronectin, and inflammatory cytokines, among others) and deficiency of vasodilators (prostacyclin and nitric oxide) have been demonstrated [13].

In recent years, new interesting research has been carried out, including studies in which the impact of seasonality on the levels of micronutrients was found [41], or studies of new strategies for compensating selenium deficiencies, as described above. We believe that our study and these new directions of research can help improve predicting and treating pregnancy-induced hypertension.

## 5. Conclusions

In this prospective study, lower serum selenium levels in early healthy pregnancy were associated with the higher risk of pregnancy-induced hypertension and showed high prognostic indicators. Serum Se levels in early healthy pregnancy served as a risk marker for this dangerous disease.

Our results show that the measurement of this element serum level in early pregnancy can be included in diagnostics for identifying women at risk of pregnancy-induced hypertension.

The results of our study can be applied in practice; this microelement level measurement is an accessible and inexpensive test.

In our study, statistically significant changes in this micronutrient serum levels in overweight/obese women and in smokers strongly influence the estimated odds ratios of pregnancy-induced hypertension. The structure of the studied populations, in terms of BMI and smoking, may cause discrepancies between studies. The mechanisms as a result of which obesity increases the risk of various diseases are very complex, but the role of Se in these mechanisms should be investigated.

Our results may suggest that it should be attempted to balance the Se level in women in early pregnancy and before pregnancy. Attention should be paid to the latest research related to modern methods of compensating this micronutrient deficiencies.

This study may help to better understand the causes of pregnancy-induced hypertension and to improve the effectiveness of treatment.

## Figures and Tables

**Table 1:** The clinical characteristics of the normotensive controls and cases of pregnancy-induced hypertension.

Characteristics	Controls (n = 363) *	Cases (n = 121) *	*p* **
	Mean (SD); Median or n (%)	Mean (SD); Median or n (%)	
Maternal age (years)	35.1 (4.0); 36.0	35.1 (4.2); 36.0	0.907
Maternal age (range)	(22–45)	(19–45)	
Primiparous	141 (38.84%)	56 (46.28%)	0.149
GDM at present pregnancy •	73 (20.11%)	23 (19.01%)	0.792
ART ••	18 (4.96%)	11 (9.09%)	0.097
Pack-years during recruitment	19.3 (32.5); 6.6	21.2 (32.3); 8.8	0.748
Women who had never smoked	302 (83.20%)	92 (76.03%)	0.080
Multivitamins in II-III trimester	184 (50.69%)	50 (41.32%)	0.074
Pre-pregnancy BMI (kg/m^2^)	25.0 (4.4); 24.4	26.8 (5.4); 26.5	0.003
Pre-pregnancy BMI (range)	(16.5–39.4)	(18.2–42.9)	
Gestational age at recruitment (weeks)	12.3 (0.8); 12.0	11.6 (0.8); 11.0	1.97 × 10^−16^
Gestational age at recruitment (range)	(10–14)	(10–14)	
Education levels (available data)	305	105	0.042
higher	201 (65.90%)	57 (54.29%)	
secondary	76 (24.92%)	28 (26.67%)	
vocational	26 (8.53%)	17 (16.19%)	
elementary	2 (0.66%)	3 (2.86%)	
Financial status (available data) ***	141	63	0.002
lower levels	14 (9.93%)	5 (7.94%)	
medium level	32 (22.70%)	26 (41.27%)	
higher levels	95 (67.38%)	32 (50.79%)	
Place of residence (available data)	362	120	0.585
countryside	110 (30.39%)	30 (25.00%)	
town <50,000 inhabitants	104 (28.73%)	35 (29.17%)	
big city >50,000 inhabitants	148 (40.88%)	56 (46.67%)	
Delivery			
Gestational age at delivery (weeks)	38.7 (1.8); 39.0	38.0 (2.6); 39.0	0.011
Newborn birthweight (g)	3385.3 (546.8); 3400.0	3113.1 (785.4); 3150.0	0.0003

* Normotensive controls and cases of pregnancy-induced hypertension (PIH); ** The Mann-Whitney U test was used for comparisons of continuous variables (*p*-value <0.05 was assumed to be significant (medians were compared) and the Pearson chi-square test was used for categorical variables comparisons (*p*-value <0.05 was assumed to be significant); • GDM: gestational diabetes mellitus; •• ART: assisted reproductive technology; *** Financial status was assessed according to a 5 Lickert′s scale (see in Method section).

**Table 2:** Serum Se levels (in the 10–14th gestational week) in the groups and subgroups.

Groups and Subgroups		Serum Se Levels * (µg/L)			*p* ***
	n	Mean	Range: Min-Max	Median	
**Whole cohort**					
Normotensive controls	363	62.89	41.14–125.54	62.02	
Cases of PIH **	121	57.51	40.91–74.36	57.40	2.59 × 10^−10^
**Pre-pregnancy BMI categories**					
BMI 18.5–24.99 kg/m^2^	265	62.23	40.91–90.14	61.57	
BMI ≥25 kg/m^2^	211	60.27	44.39–125.54	58.91	7.00 × 10^−4^
**Smoking categories**					
Women who had never smoked	394	61.91	44.39–125.54	61.22	
Smokers at the time of recruitment	32	57.74	40.91–70.03	57.62	1.80 × 10^−2^
**Subgroup #**					
Normotensive Controls	184	63.58	48.35–90.14	62.80	
Cases of PIH **	44	57.69	44.63–70.13	58.65	8.93·× 10^−7^

**#** Subgroup of women who had never smoked with normal pre-pregnancy BMI; * Se levels in serum in the 10–14th gestational week; ** PIH: pregnancy-induced hypertension; *** *p*-value obtained using the Mann-Whitney U test, *p* < 0.05 was assumed to be significant (medians were compared); # normal pre-pregnancy BMI: body mass index 18.50–24.99 kg/m^2^.

**Table 3:** The risk of pregnancy-induced hypertension for serum Se levels in univariate and multivariate logistic regression.

Risk of Pregnancy-Induced Hypertension									
Quartile	Se (µg/L) !	Cases •	Controls	OR *	(CI 95%)	*p* ***	AOR **	(CI 95%)	*p* ***
Whole cohort									
Q_1_	40.91–55.83	47	74	5.28	(2.67–10.43)	1.73 × 10^−6^	3.96	(1.87–8.42)	0.0003
Q_2_	55.83–60.99	41	80	4.26	(2.14–8.47)	1.63·× 10^−5^	3.92	(1.84–8.32)	0.0004
Q_3_	60.99–65.82	20	101	1.65	(0.78–3.48)	0.193	1.85	(0.80–4.28)	0.150
Q_4_	65.82–125.54	13	108	1			1		
Subgroup #									
Q_1_	44.63–57.68	20	37	14.87	(3.28–67.43)	4.68·× 10^−4^	15.34	(2.73–86.31)	0.002
Q_2_	57.68–61.65	16	41	10.73	(2.34–49.29)	0.002	12.38	(2.28–67.34)	0.004
Q_3_	61.65–66.60	6	51	3.24	(0.62–16.76)	0.162	4.12	(0.61–27.73)	0.146
Q_4_	66.60–90.14	2	55	1			1		

# Subgroup of women who had never smoked with the normal pre-pregnancy BMI: body mass index 18.50–24.99 kg/m^2^; ! Border values are included in the lower quartile; • Cases: pregnancy-induced hypertension (PIH); Controls: normotensive women; * OR: crude odds ratio calculated in univariate logistic regression; ** AOR: adjusted odds ratio calculated in multivariate logistic regression, after adjusting for the gestational age at recruitment, rate of gestational weight gain (calculated for the whole gestation), family history of chronic hypertension, mother′s education <12 years (in the subgroup, the pre-pregnancy BMI was excluded); CI: confidence intervals; *** *p*-value obtained using the Wald test, *p* < 0.05 was assumed to be significant.

**Table 4:** Prediction indicators of pregnancy-induced hypertension for Se levels in serum in the 10–14th gestational week in test sets of logistic regression and neural network.

Method and Set	Prediction Indicators of Pregnancy-Induced Hypertension for Se Levels *					
	DR	PPV	NPV	LR+	ACC	AUC
**Test set**						
Logistic regression (FPR 5%)	37.50%	69.23%	82.95%	7.219	81.19%	0.814
Logistic regression (FPR 10%)	45.83%	57.89%	84.15%	4.411	79.21%	0.814
Neural network (FPR 5%)	29.17%	58.33%	80.90%	4.492	78.22%	0.761
Neural network (FPR 10%)	29.17%	46.67%	80.23%	2.807	75.25%	0.761

* Se levels were measured in serum from the 10–14th gestational week (µg/L); FPR 5% and 10%-fixed false positive rates; DR—detection rate (sensitivity at a fixed FPR); PPV—positive predictive value; NPV—negative predictive value; LR+ positive likelihood ratio; ACC—accuracy; AUC—area under curve ROC.

## Supplementary Materials

The following are available online at https://www.mdpi.com/2072-6643/11/5/1028/s1, Table S1: Complete characteristics of serum Se levels in the whole cohort, Table S2: Prediction indicators of pregnancy-induced hypertension for serum selenium levels in the 10–14th gestational week, in training sets of logistic regression and neural network methods, Figure S1: The risk of pregnancy-induced hypertension for selenium levels in the 10–14th pregnancy week in the subgroup of women who had never smoked with normal pre-pregnancy BMI. The graph illustrates the changes in the odds ratios (OR) of pregnancy-induced hypertension (PIH), calculated on a sliding window with respect to the changes in the selenium levels in serum in 10–14 pregnancy week. The window width adopted was 50 observations. The (light blue) points correspond to the odds ratios of pregnancy-induced hypertension in a window containing a fixed number of neighboring cases, whose center is for a selenium level value. The (red) curve represents the risk profile smoothed with the Lowess method. The horizontal (black) line marks is the reference line for OR = 1.0; the points above this line indicate an increased risk, and the points below this line correspond to a reduction in risk.

## References

[1] Choi R., Sun J., Yoo H., Kim S., Cho Y.Y., Kim H.J., Kim S.W., Chung J.H., Oh S.-Y., Lee S.-Y. (2016). A Prospective Study of Serum Trace Elements in Healthy Korean Pregnant Women. Nutrients.

[2] Kieliszek M. (2019). Selenium–Fascinating Microelement, Properties and Sources in Food. Molecules.

[3] Kieliszek M., Błażejak S. (2016). Current Knowledge on the Importance of Selenium in Food for Living Organisms: A Review. Molecules.

[4] Rayman M.P. (2012). Selenium and human health. Lancet.

[5] Santi C., Bagnoli L. (2017). Celebrating Two Centuries of Research in Selenium Chemistry: State of the Art and New Prospective. Molecules.

[6] Estevam E.C., Witek K., Faulstich L., Nasim M.J., Latacz G., Domínguez-Álvarez E., Kieć-Kononowicz K., Demasi M., Handzlik J., Jacob C. (2015). Aspects of a Distinct Cytotoxicity of Selenium Salts and Organic Selenides in Living Cells with Possible Implications for Drug Design. Molecules.

[7] Gandin V., Fernandes A.P. (2015). Metal- and Semimetal-Containing Inhibitors of Thioredoxin Reductase as Anticancer Agents. Molecules.

[8] Maggini S., Pierre A., Calder P.C. (2018). Immune Function and Micronutrient Requirements Change over the Life Course. Nutrients.

[9] Park K., Seo E. (2016). Association between Toenail Mercury and Metabolic Syndrome Is Modified by Selenium. Nutrients.

[10] Reszka E., Jablonska E., Gromadzinska J., Wasowicz W. (2012). Relevance of selenoprotein transcripts for selenium status in humans. Genes Nutr..

[11] Qazi I.H., Angel C., Yang H., Pan B., Zoidis E., Zeng C.-J., Han H., Zhou G.-B. (2018). Selenium, Selenoproteins, and Female Reproduction: A Review. Molecules.

[12] Vanderlelie J., Perkins A.V.A. (2011). Selenium and preeclampsia: A global perspective. Pregnancy Hypertens..

[13] Rayman M.P., Searle E., Kelly L., Johnsen S., Bodman-Smith K., Bath S.C., Mao J., Redman C.W.G. (2014). Effect of selenium on markers of risk of pre-eclampsia in UK pregnant women: A randomised, controlled pilot trial. Br. J. Nutr..

[14] Duhig K., Vandermolen B., Shennan A. (2018). Recent advances in the diagnosis and management of pre-eclampsia. F1000Research.

[15] Myatt L., Webster R.P. (2009). Vascular biology of preeclampsia. J. Thromb. Haemost..

[16] Anton L., Olarerin-George A.O., Hogenesch J.B., Elovitz M.A. (2015). Placental expression of miR-517a/b and miR-517c contributes to trophoblast dysfunction and preeclampsia. PLoS ONE.

[17] Haque M.M., Moghal M.M.R., Sarwar M.S., Anonna S.N., Akter M., Karmakar P., Ahmed S., Sattar M.A., Islam M.S. (2016). Low serum selenium concentration is associated with preeclampsia in pregnant women from Bangladesh. J. Trace Elem. Med. Biol..

[18] Farzin L., Sajadi F. (2012). Comparison of serum trace element levels in patients with or without pre-eclampsia. J. Res. Med. Sci..

[19] Mistry H.D., Kurlak L.O., Williams P.J., Ramsay M.M., Symonds M.E., Broughton Pipkin F. (2010). Differential expression and distribution of placental glutathione peroxidases 1, 3 and 4 in normal and preeclamptic pregnancy. Placenta.

[20] Malinova M., Paskaleva V. (2013). Selenium and glutathione peroxidase in patients with preeclampsia. Akush Ginekol (Sofiia).

[21] da Silva A.C., Martins-Costa S.H., Valério E.G., Lopes Ramos J.G. (2017). Comparison of serum selenium levels among hypertensive and normotensive pregnant women. Hypertens. Pregnancy.

[22] Rayman M.P., Bath S.C., Westaway J., Williams P., Mao J., Vanderlelie J.J., Perkins A.V., Redman C.W.G. (2015). Selenium status in U.K. pregnant women and its relationship with hypertensive conditions of pregnancy. Br. J. Nutr..

[23] Ghaemi S.Z., Forouhari S., Dabbaghmanesh M.H., Sayadi M., Bakhshayeshkaram M., Vaziri F., Tavana Z. (2013). A prospective study of selenium concentration and risk of preeclampsia in pregnant Iranian women: A nested case-control study. Biol. Trace Elem. Res..

[24] Mistry H.D., Gill C.A., Kurlak L.O., Seed P.T., Hesketh J.E., Méplan C., Schomburg L., Chappell L.C., Morgan L., Poston L. (2015). Association between maternal micronutrient status, oxidative stress, and common genetic variants in antioxidant enzymes at 15 weeks’ gestation in nulliparous women who subsequently develop preeclampsia. Free Radic. Biol. Med..

[25] Basu A., Yu J.Y., Jenkins A.J., Nankervis A.J., Hanssen K.F., Henriksen T., Lorentzen B., Garg S.K., Menard M.K., Hammad S.M. (2015). Trace elements as predictors of preeclampsia in type 1 diabetic pregnancy. Nutr. Res..

[26] Stefanowicz F.A., Talwar D., O’Reilly D.S.J., Dickinson N., Atkinson J., Hursthouse A.S., Rankin J., Duncan A. (2013). Erythrocyte selenium concentration as a marker of selenium status. Clin. Nutr..

[27] Rosenberg-Hasson Y., Hansmann L., Liedtke M., Herschmann I., Maecker H.T. (2014). Effects of serum and plasma matrices on multiplex immunoassays. Immunol. Res..

[28] Katz O., Paz-Tal O., Lazer T., Aricha-Tamir B., Mazor M., Wiznitzer A., Sheiner E. (2012). Severe pre-eclampsia is associated with abnormal trace elements concentrations in maternal and fetal blood. J. Matern. Fetal. Neonatal. Med..

[29] Xu M., Guo D., Gu H., Zhang L., Lv S. (2016). Selenium and Preeclampsia: A Systematic Review and Meta-analysis. Biol. Trace Elem. Res..

[30] Salles A.M.R., Galvao T.F., Silva M.T., Motta L.C.D., Pereira M.G. (2012). Antioxidants for preventing preeclampsia: A systematic review. Sci. World J..

[31] Stranges S., Laclaustra M., Ji C., Cappuccio F.P., Navas-Acien A., Ordovas J.M., Rayman M., Guallar E. (2010). Higher selenium status is associated with adverse blood lipid profile in British adults. J. Nutr..

[32] Lubiński J., Marciniak W., Muszynska M., Jaworowska E., Sulikowski M., Jakubowska A., Kaczmarek K., Sukiennicki G., Falco M., Baszuk P. (2018). Correction: Serum selenium levels and the risk of progression of laryngeal cancer. PLoS ONE.

[33] Jaworska K., Gupta S., Durda K., Muszyńska M., Sukiennicki G., Jaworowska E., Grodzki T., Sulikowski M., Waloszczyk P., Woloszczyk P. (2013). A low selenium level is associated with lung and laryngeal cancers. PLoS ONE.

[34] Lener M., Muszyńska M., Jakubowska A., Jaworska-Bieniek K., Sukiennicki G., Kaczmarek K., Durda K., Gromowski T., Serrano-Fernández P., Kładny J. (2015). Selenium as a marker of cancer risk and of selection for control examinations in surveillance. Contemp. Oncol. (Pozn.).

[35] Nwagha U.I., Ogbodo S.O., Nwogu-Ikojo E.E., Ibegbu D.M., Ejezie F.E., Nwagha T.U., Dim C.C. (2011). Copper and selenium status of healthy pregnant women in Enugu, southeastern Nigeria. Niger. J. Clin. Pract..

[36] Fan Y., Zhang C., Bu J. (2017). Relationship between Selected Serum Metallic Elements and Obesity in Children and Adolescent in the U.S. Nutrients.

[37] Mao J., Vanderlelie J.J., Perkins A.V., Redman C.W.G., Ahmadi K.R., Rayman M.P. (2016). Genetic polymorphisms that affect selenium status and response to selenium supplementation in United Kingdom pregnant women. Am. J. Clin. Nutr..

[38] Wu F., Tian F.-J., Lin Y. (2015). Oxidative Stress in Placenta: Health and Diseases. Biomed. Res. Int..

[39] Khera A., Vanderlelie J.J., Perkins A.V. (2013). Selenium supplementation protects trophoblast cells from mitochondrial oxidative stress. Placenta.

[40] Watson M., van Leer L., Vanderlelie J.J., Perkins A.V. (2012). Selenium supplementation protects trophoblast cells from oxidative stress. Placenta.

[41] Liang C.-M., Wu X.-Y., Huang K., Yan S.-Q., Li Z.-J., Xia X., Pan W.-J., Sheng J., Tao Y.-R., Xiang H.-Y. (2019). Trace element profiles in pregnant women’s sera and umbilical cord sera and influencing factors: Repeated measurements. Chemosphere.

