# Supplementary material for: Serum Selenium Level in Early Healthy Pregnancy as a Risk Marker of Pregnancy Induced Hypertension
Source: Nutrients. 2019 May 8;11(5):1028. doi: 10.3390/nu11051028 (PMC6566672; doi:10.3390/nu11051028)
Supplement: Supplementary file 1 [file nutrients-11-01028-s001.zip › Table S2.docx]

**Table S2.** Prediction indicators of pregnancy induced hypertension for serum selenium levels in the 10-14 gestational week; in training sets of logistic regression and neural network methods

| **Prediction indicators of pregnancy induced hypertension for Se levels *** | | | | | | |
| --- | --- | --- | --- | --- | --- | --- |
| **Method and set** | **DR** | **PPV** | **NPV** | **LR +** | **ACC** | **AUC** |
| **Training set** |  |  |  |  |  |  |
| Logistic regression (FPR 5%) | 17.53% | 53.13% | 77.21% | 3.342 | 75.20% | 0.659 |
| Logistic regression (FPR 10%) | 20.62% | 40.82% | 76.95% | 2.033 | 72.32% | 0.659 |
|  |  |  |  |  |  |  |
| Neural network (FPR 5%) | 11.34% | 42.31% | 75.91% | 2.162 | 73.63% | 0.644 |
| Neural network (FPR 10%) | 17.53% | 36.96% | 76.26% | 1.728 | 71.54% | 0.644 |

* Selenium levels were measured in serum from 10-14 gestational week (µg/L); FPR 5% and 10% - fixed false positive rates; DR- detection rate (sensivity at a fixed FPR); PPV- positive predictive value; NPV- negative predictive value; LR+ positive likelihood ratio; ACC accuracy; AUC area under curve ROC
