# Supplementary material for: Serum Selenium Level in Early Healthy Pregnancy as a Risk Marker of Pregnancy Induced Hypertension
Source: Nutrients. 2019 May 8;11(5):1028. doi: 10.3390/nu11051028 (PMC6566672; doi:10.3390/nu11051028)
Supplement: Supplementary file 1 [file nutrients-11-01028-s001.zip › Figure S1.docx]

**
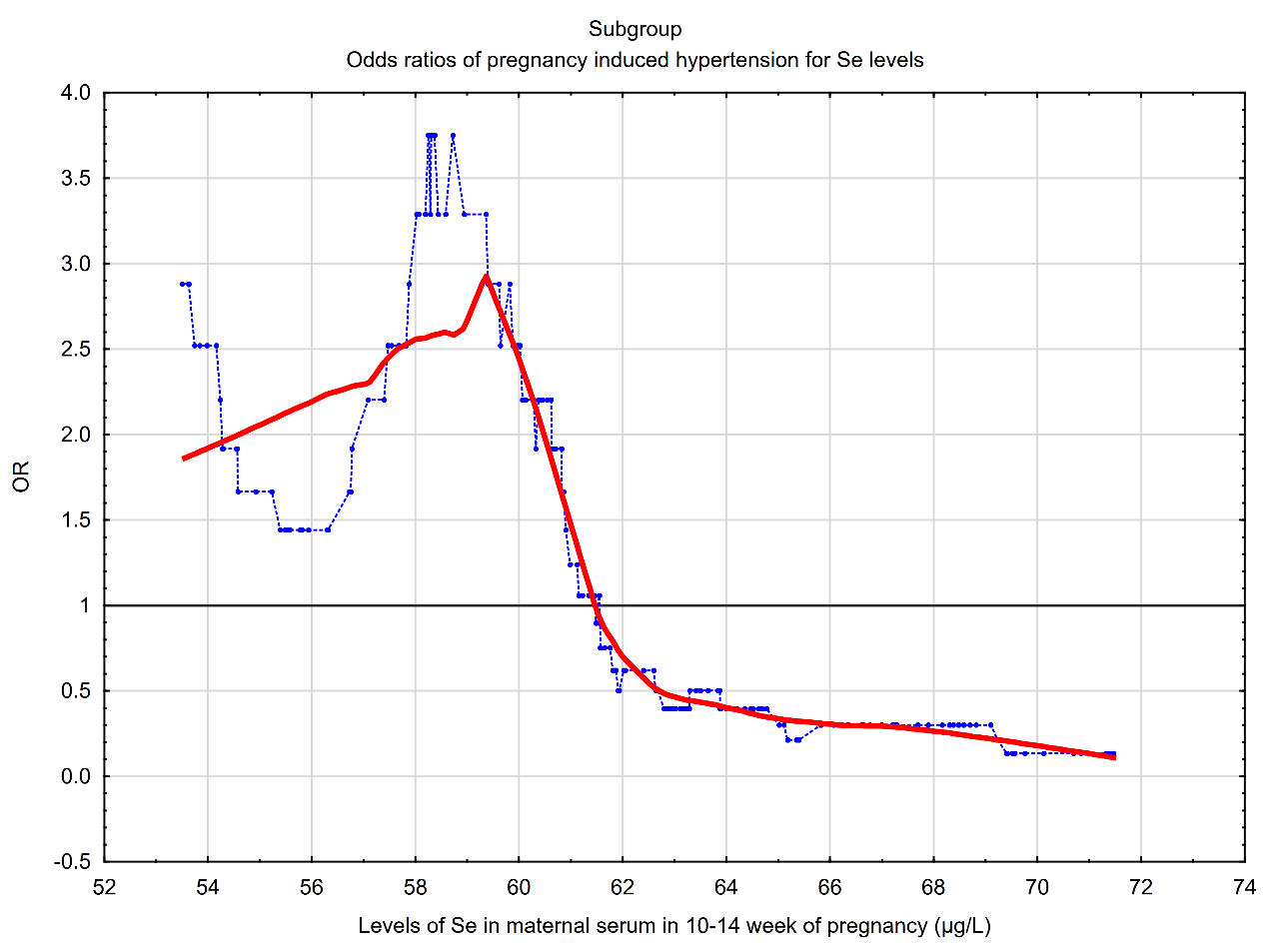
**

**Figure S1.** The risk of pregnancy-induced hypertension for selenium levels in the 10–14th pregnancy week in the subgroup of women who had never smoked with normal pre-pregnancy BMI. The graph illustrates the changes in the odds ratios (OR) of pregnancy-induced hypertension (PIH), calculated on a sliding window with respect to the changes in the selenium levels in serum in 10-14 pregnancy week. The window width adopted was 50 observations. The (light blue) points correspond to the odds ratios of pregnancy-induced hypertension in a window containing a fixed number of neighboring cases, whose center is for a selenium level value. The (red) curve represents the risk profile smoothed with the Lowess method. The horizontal (black) line marks is the reference line for OR = 1.0; the points above this line indicate an increased risk, and the points below this line correspond to a reduction in risk.
