# Supplementary material for: Serum Selenium Level in Early Healthy Pregnancy as a Risk Marker of Pregnancy Induced Hypertension
Source: Nutrients. 2019 May 8;11(5):1028. doi: 10.3390/nu11051028 (PMC6566672; doi:10.3390/nu11051028)
Supplement: Supplementary file 1 [file nutrients-11-01028-s001.zip › Table S1.docx]

**Table S1.** Complete characteristics of serum Se levels in the whole cohort.

| **Serum Se levels * (µg/L)** | | | | | | | | | |
| --- | --- | --- | --- | --- | --- | --- | --- | --- | --- |
| **Groups **** | **n** | **Mean** | **SD** | **Min.** | **Max.** | **Q25** | **Median** | **Q75** | **p ***** |
| Normotensive controls | 363 | 62.89 | 8.7 | 41.14 | 125.54 | 57.57 | 62.02 | 67 | 2.59·10^-10^ |
| Cases of PIH ** | 121 | 57.51 | 6.54 | 40.91 | 74.36 | 52.83 | 57.4 | 61.58 |  |
| Whole cohort | 484 | 61.55 | 8.53 | 40.91 | 125.54 | 55.85 | 61.06 | 65.87 |  |

* Se levels were measured in serum from 10-14 gestational week; ** PIH: pregnancy induced hypertension;
*** p-value obtained using the Mann-Whitney test; p < 0.05 was assumed to be significant
