# Supplementary figures and images for: Serum Selenium Level in Early Healthy Pregnancy as a Risk Marker of Pregnancy Induced Hypertension
Source: Nutrients. 2019 May 8;11(5):1028. doi: 10.3390/nu11051028 (PMC6566672; doi:10.3390/nu11051028)

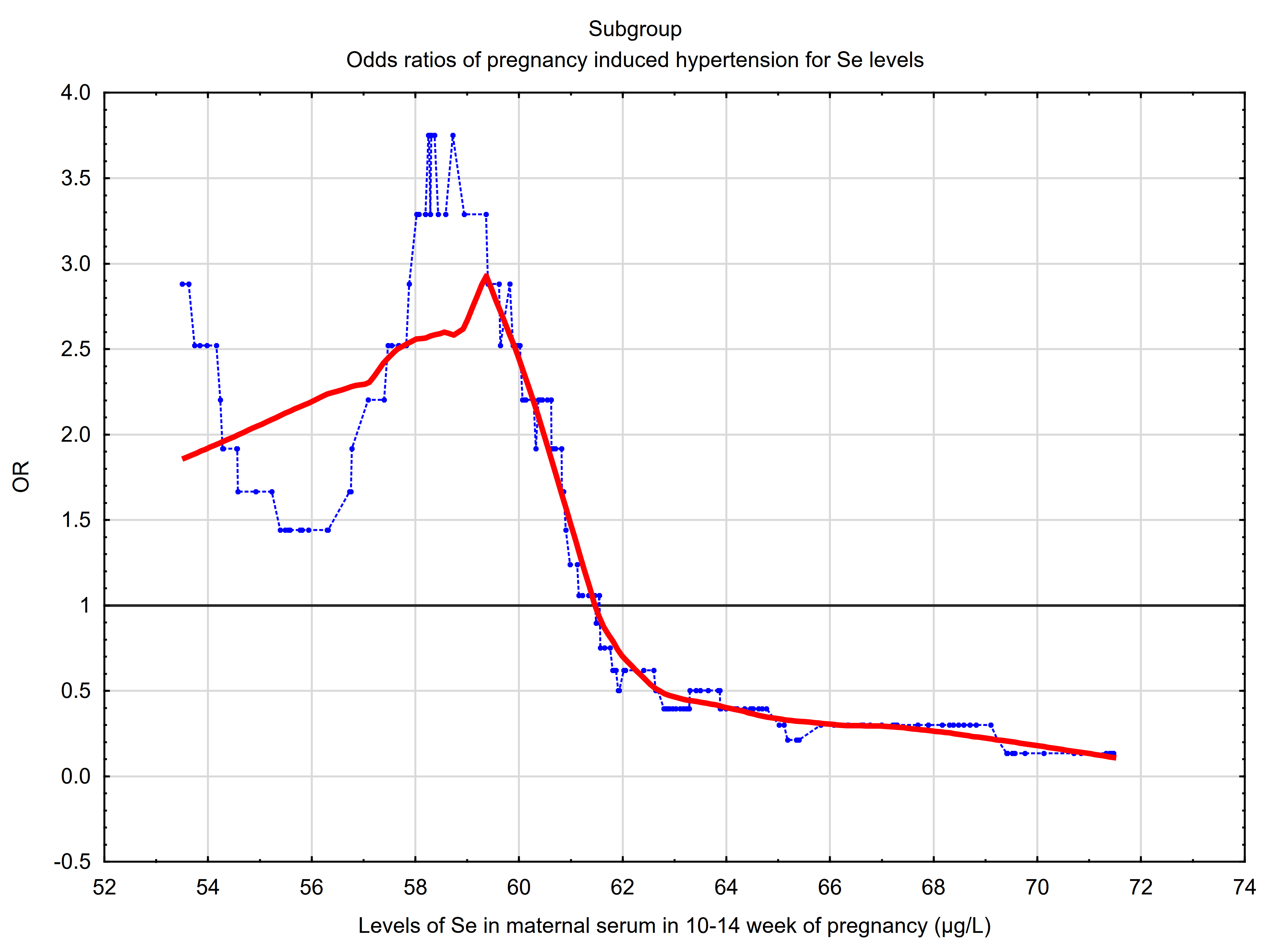

Supplement: Supplementary file 1 [file nutrients-11-01028-s001.zip › Fig. S1.tif]
